# Mechanistic Study on the Baseline Drift Phenomenon of Piezoelectric Pressure Sensors When Measuring Blast Waves

**DOI:** 10.3390/s26082430

**Published:** 2026-04-15

**Authors:** Yaolong Li, Jie Zhu, Liqiang Chen, Qianqian Cheng, Hailong Hui, Jin Li, Jun Yang, Zutang Wu

**Affiliations:** National Key Laboratory of Intense Pulsed Radiation Simulation and Effect, Northwest Institute of Nuclear Technology, Xi’an 710024, China; liyaolong@nint.ac.cn (Y.L.); zhujie@nint.ac.cn (J.Z.); chenliqiang@nint.ac.cn (L.C.); chengqianqian@nint.ac.cn (Q.C.); yangjun@nint.ac.cn (J.Y.); wuzutang@nint.ac.cn (Z.W.)

**Keywords:** explosion, blast wave measurement, piezoelectric pressure sensor, baseline drift phenomenon, light radiation, thermal shock, mechanism analysis

## Abstract

Piezoelectric pressure sensors are commonly used as blast wave pressure sensors in explosion testing. Accurate measurement of blast wave overpressure is of great significance. In explosion testing, piezoelectric pressure sensors exhibit a baseline drift phenomenon. This paper analyzes the mechanism of the baseline drift phenomenon observed in explosion testing through experiments and simulation, identifying the mechanism behind it. From an experimental perspective, it is determined that the thermal effect of light induces the baseline drift phenomenon. Furthermore, modeling and simulation of the piezoelectric pressure sensor using COMSOL 6.2 Multiphysics software confirms that the photothermal effect causes changes in the temperature field within the sensor’s internal structure, which in turn brings the thermal stress. The thermal stress superimposes on the output of piezoelectric pressure sensors. This is the fundamental cause of the baseline drift phenomenon in piezoelectric pressure sensors. This research provides a crucial foundation for understanding the mechanisms by which explosions affect piezoelectric pressure sensors.

## 1. Introduction

When explosive materials detonate, the high-temperature and high-pressure combustion products rapidly compress the surrounding medium, generating a blast wave. Blast waves present a significant hazard to various assets, such as human life, infrastructure, and military vehicles [1]. The blast wave overpressure is an important indicator for measuring the intensity of an explosion event. It is also a crucial basis for evaluating the destructive performance and injury effects of various weapons and equipment [2]. Therefore, accurately measuring the blast wave overpressure is of great significance in the field of blast physics and munitions engineering.

At present, blast wave testing technology has developed multiple methods, including the electrometric method, equivalent target plate method, biological experiment method and optical measurement method [2]. Among these, the electrometric method characterizes the time-dependent variation in blast wave overpressure by recording signals collected by pressure sensors. This method provides intuitive measurement results, clearly displaying parameters such as the peak value of blast wave overpressure, duration of action, and specific impulse. It is named the “electrometric method” because electric sensors were the first ones to be developed. With technological advancement, fiber optic pressure sensors have also emerged, but there are currently few commercial products available.

Commonly used electrometric sensors can be divided into piezoresistive pressure sensors and piezoelectric pressure sensors. Piezoresistive pressure sensors exhibit good low-frequency characteristics, yet they are highly susceptible to external factors such as temperature and light. As a result, they are generally not used in damage conditions involving intense flames, significant temperature changes, or strong ionized fields. Although piezoelectric pressure sensors are inferior to piezoresistive ones in terms of zero-frequency and low-frequency characteristics, they offer excellent dynamic performance, high stability, and low sensitivity to external interference. Therefore, in the fields of explosion testing and damage assessment, piezoelectric pressure sensors are the most commonly used to measure the blast wave overpressure [3].

In Ref. [4], the influence of strong light on the performance of a piezoresistive pressure sensor was investigated. Its experimental results show that the output of piezoresistive pressure sensors is proportional to light intensity. Simulation results further confirm that when light irradiates the exposed silicon sensitive surface of the piezoresistive sensor, the photoelectric effect occurs, and the photocurrent has a linear relationship with light intensity. Therefore, the output of piezoresistive pressure sensors is proportional to light intensity. Ref. [1] mentions that piezoelectric sensors have no photosensitivity. However, Ref. [5] (pp. 223–227) detected a baseline drift phenomenon in piezoelectric pressure sensors when measuring explosive blast waves during experiments, and attributed this phenomenon to the photoelectric effect of the explosion. In fact, the sensitive surface of a piezoelectric sensor is usually made of steel (with a work function of approximately 4.3 eV to 4.8 eV). Ultraviolet light with wavelengths of approximately 276 nm or shorter can induce the photoelectric effect on a steel surface. However, in chemical explosions, the light emission can be approximated as blackbody radiation. Studies on explosive-driven shock waves have confirmed that the shock fronts radiate a blackbody spectrum [6], and measurements on liquid explosives (nitromethane, etc.) have yielded detonation temperatures of 3300–3500 K, with emissivities close to that of a true blackbody [7]. At such typical temperatures (approximately 3300–5000 K), the peak spectral radiance, calculated by Wien‘s displacement law, lies within the visible to near-infrared range (approximately 580–880 nm). Consequently, the proportion of radiation energy in the deep ultraviolet region (<300 nm) is extremely low, making the photoelectric effect highly unlikely. Therefore, the baseline drift phenomenon cannot be attributed to photoelectric effects.

Nevertheless, the occurrence of the baseline drift phenomenon of piezoelectric pressure sensors during explosion tests is not uncommon. For instance, the baseline drift phenomenon has been reported in Refs. [8,9,10] The authors also observed this baseline drift phenomenon in their own explosion experiments. Section 2 will give an example of the baseline drift phenomenon.

The baseline drift phenomenon can affect the interpretation of the peak value of blast wave overpressure and the interpretation of positive pressure duration. Based on experiments and multiphysics field simulations, this paper aims to reveal the baseline drift phenomenon that occurs when measuring explosive blast waves using piezoelectric pressure sensors.

It should be noted that the piezoelectric pressure sensors referred to in this paper are quartz-based sensors. Sensors based on piezoelectric ceramics or PVDF are not discussed, as they would introduce a pyroelectric effect, which would complicate the analysis.

The paper is organized as follows. In Section 2, an explosion test was carried out to bring out the baseline drift phenomenon of piezoelectric pressure. By analyzing the results, the cause of the baseline drift phenomenon is preliminarily identified as the light generated during the explosion. Further experiments are conducted to confirm that light is indeed the cause of the baseline drift phenomenon. Section 3 utilizes COMSOL 6.2 Multiphysics software to reveal how light induces internal changes in piezoelectric sensors, leading to the baseline drift phenomenon. Section 4 is the discussion, and Section 5 presents the conclusion.

## 2. The Baseline Drift Phenomenon of Piezoelectric Pressure Sensors When Measuring Blast Waves

**Figure 1:**
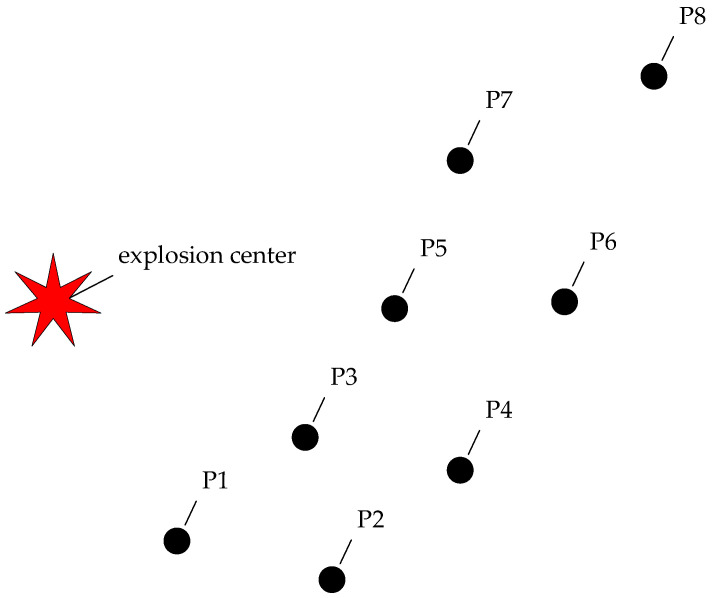
The top-view schematic diagram of the experiment.

To study the damaging effects of explosives on objects, a field experiment with a 1 kg TNT charge was conducted. The top-view schematic diagram of the experiment is shown in Figure 1. The blast wave produced by the charge was recorded by eight gauging points, with the names P1 to P8. The explosive charge was placed on a wooden stand, 1.5 m above the ground. The horizontal distances from the gauging points to the explosion center were 3.43 m, 5.09 m, 3.95 m, 5.78 m, 5.89 m, 8.67 m, 8.41 m, 12.25 m, respectively. The distances were arranged in an increasing alternating pattern.

**Figure 2:**
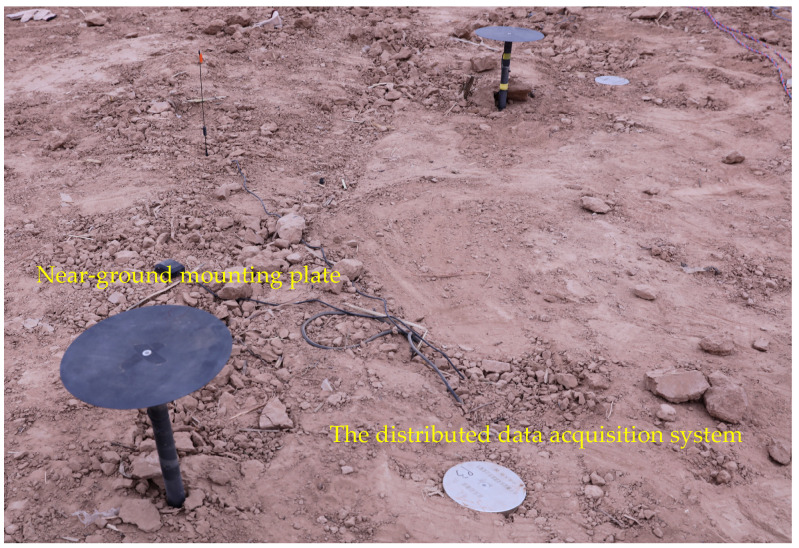
The two measurement methods are employed at a single gauging point.

Two methods were employed to measure the blast wave at each gauging point, as illustrated in Figure 2. In the first method, a near-ground mounting plate was utilized. The Kistler 603CBA00014 type IEPE piezoelectric pressure sensor (the range is 1.4 MPa) was placed at the center of this plate, which was positioned approximately 20 cm above the ground. Kistler is a leading manufacturer in the field of piezoelectric pressure sensors, with its headquarters located in Winterthur, Switzerland. A low-noise cable connected the sensor and the data acquisition system, which was located 100 m away from the explosion point. In the second method, a distributed data acquisition system was placed beside the near-ground plate. The sensor was integrated at the center of this instrumentation. The instrumentation has functionalities including power supply, data storage, and triggering. Each method was equipped with a time synchronization capability. The data presented in this paper were all obtained using the first method.

**Figure 3:**
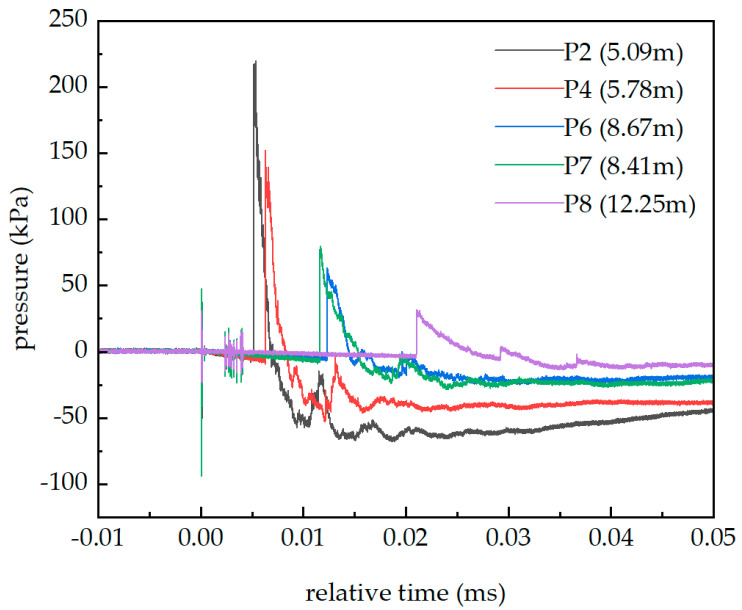
The result of the blast wave overpressure in the 1 kg TNT explosion experiment.

Figure 3 presents the blast wave measurement results. Data from P1, P3, and P5 are missing. The baseline drift phenomenon can be observed in all five curves. The amplitude of pressure peaks decreases as the distance from the explosion center increases.

**Figure 4:**
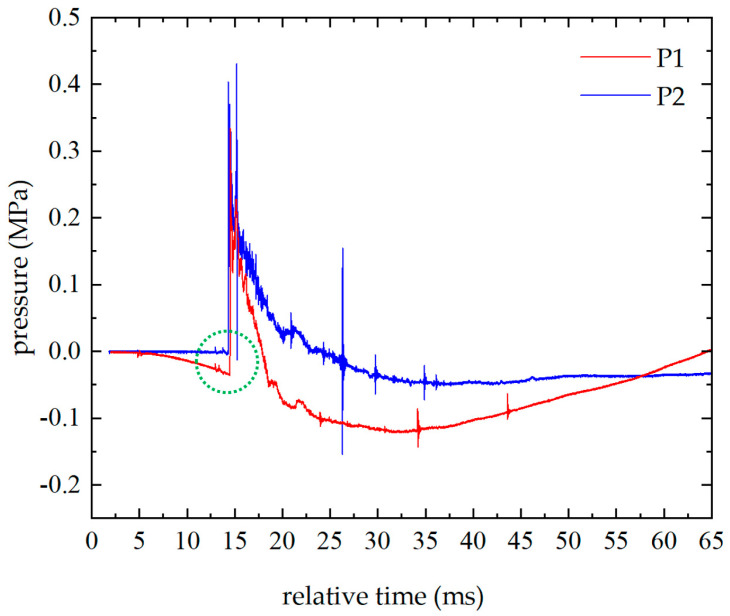
Comparison of the overpressure of two piezoelectric pressure sensors while the P2’s sensitive diaphragm is attached with black PVC film.

When conducting blast wave overpressure tests in the explosion test, the blast wave is often accompanied by parasitic effects such as high temperature, seismic waves, and mechanical impacts. The various physical fields act together on the sensor, causing the measured signal to be distorted [3]. By analyzing the mechanism of the parasitic effects and the sequence of their arrival times, the extent of their influence can be further determined. For example, the seismic waves from the middle and far fields reach the sensor measurement point position earlier than the blast wave, and a small pulse can be observed before the blast wave signal (this can be seen in Figure 4, marked with a green dot dash circle). For instance, the electromagnetic waves generated by the explosion propagate at nearly the speed of light and can couple into the test system cable. Observing Figure 3, a high electromagnetic pulse was generated during the explosion and coupled into the test system, which is an electromagnetic signal-induced parasitic effect. At 3–5 ms after zero, there is also an electromagnetic radiation signal coupling into the test system, which is basically consistent with the description of the initial explosion electromagnetic radiation signal described in Ref. [11].

From Figure 3, it can be observed that the baseline drift phenomenon of the piezoelectric pressure sensor begins to occur immediately at zero time of the explosion and persists for a long time. The propagation speed of the baseline drift phenomenon is much faster than that of the blast wave. At the same time, from Figure 3, it can be found that as the explosion center distance increases, the drift phenomenon weakens, and the explosion light intensity also decreases with the increase in trade-off distance. Therefore, it can be preliminarily determined that light is the cause of the baseline drift phenomenon.

Furthermore, another explosion experiment has been carried out. Figure 4 is a comparison of the overpressure of two piezoelectric pressure sensors in the explosion experiment. On the sensitive diaphragm of P2, a 0.13 mm black polyvinyl chloride film was attached. It can be observed that after attaching the film, the baseline drift phenomenon basically disappears. This further confirms that light is the cause of the drift phenomenon.

**Figure 5:**
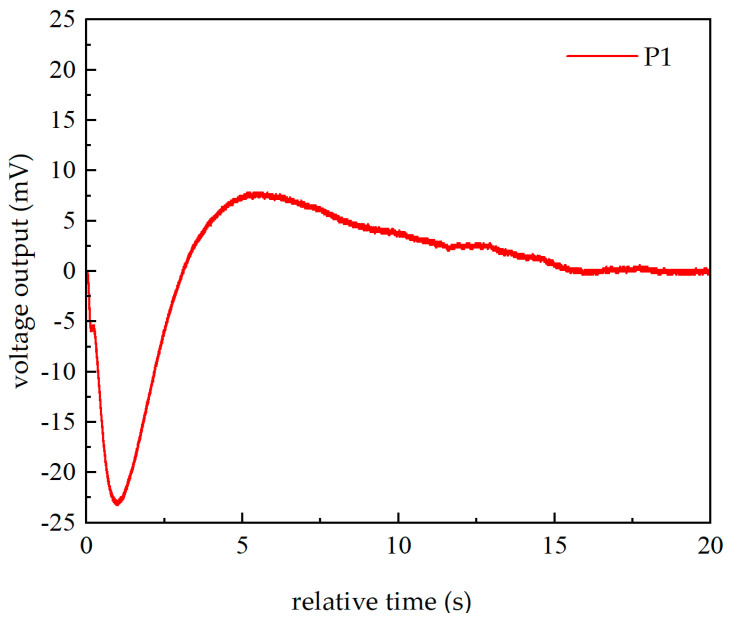
Output of a Kistler 603CBA00070 sensor under tungsten lamp illumination.

To further confirm that light is the reason, indoor experiments were conducted. A fixed tungsten lamp was used to continuously illuminate the sensitive diaphragm of the piezoelectric pressure sensor, and the sensor output results are shown in Figure 5. The type of sensor is Kistler 603CBA00070. After being illuminated by the tungsten lamp, the baseline signal of the sensor exhibits a two-stage decline, followed by an increase, and ultimately returns to zero, once again confirming that light was the cause of the baseline drift phenomenon.

When the sensor was sealed and placed in a bath, the large specific heat capacity of water could reduce the temperature rise in the sensor’s sensitive diaphragm. Under the illumination of the tungsten lamp, the sensor’s output remained basically unchanged. When the sensor’s sensitive diaphragm was illuminated by a cold light source, the output change was smaller than that when illuminated by a tungsten lamp in the air. Additionally, the sensor used was a quartz-based piezoelectric pressure sensor, and the piezoelectric crystal of this sensor did not have a pyroelectric effect. Through the above experiments and analysis, it can be determined that the thermal effect of light is the core reason for the baseline drift phenomenon of the piezoelectric sensor.

Many references (e.g., Ref [12,13,14,15,16]) have concluded that heat has an impact on the piezoelectric pressure sensors. Through the tests in this section, we conducted two explosion experiments and several small-scale tests to qualitatively demonstrate that the thermal effect of light is the cause of the baseline drift phenomenon when measuring blast waves.

## 3. Simulation Analysis

Section 2 has basically determined through experiments that the baseline drift phenomenon of the piezoelectric pressure sensor is caused by the thermal effect of light. In this section, the influence of light radiation on the piezoelectric sensor will be modeled using COMSOL Multiphysics, from a deeper perspective to reveal how the thermal radiation effect of light actually causes the baseline drift phenomenon.

### 3.1. Introduction of COMSOL Multiphysics Software

The COMSOL Multiphysics software is dedicated to multiphysics simulation analysis. It can simultaneously conduct simulations for multiple physical fields such as electromagnetism, structural mechanics, acoustics, heat transfer, piezoelectricity, etc. For the simulation of pressure sensors under light illumination, multiple physical fields, including structural mechanics, piezoelectric effect, heat transfer, and thermal expansion, need to be considered. Therefore, this paper uses COMSOL Multiphysics for simulation.

### 3.2. Modeling of Piezoelectric Pressure Sensors

**Figure 6:**
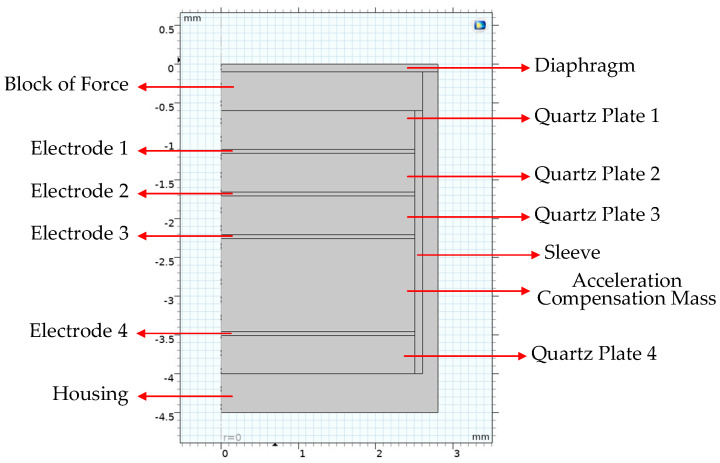
The simulation model of a piezoelectric pressure sensor in COMSOL Multiphysics.

Based on the Refs. [12,15,17], the simulation model of piezoelectric pressure sensors is established, and the piezoelectric pressure sensor model with the acceleration compensation is adopted. Considering the sensor is a two-dimensional axisymmetric structure, the two-dimensional axisymmetric model is modeled in the software, which can reduce the simulation time effectively. The simulation model of the pressure sensor in COMSOL Multiphysics is shown in Figure 6. The geometrical parameters of the simulation model are shown in Table 1, and the material properties are derived from the software’s built-in materials warehouse. The thermophysical properties of materials are mentioned by Ref. [12], as shown in Table 2. The materials are set to isotropy except for quartz. Three physical fields, solid mechanics, heat expansion and piezoelectric with two multi-physical coupling fields, thermal expansion and piezoelectricity, are established.

The structure of piezoelectric pressure sensors includes a diaphragm, a block of force, a sleeve, a housing, some piezoelectric plates and electrodes, an acceleration compensation mass, etc. The piezoelectric crystals are the core parts of the sensors, which provide piezoelectric effects. This article uses alpha quartz as a piezoelectric crystal. Quartz has high mechanical strength, good insulation, good time stability, good temperature stability, high concentration and no thermoelectric effect, but relatively small piezoelectric constants. To make the response of the sensor more linear, a preload force needs to be applied to the piezoelectric pressure sensor. The diaphragm, which is the sensitive surface, serves two purposes: it provides a preload force to the piezoelectric crystal and transmits the force to the piezoelectric crystal through the force transmission block. When the blast wave pressure load acts on the surface of the diaphragm, the force will be transmitted sequentially downward. The charge signals generated by the quartz under the pressure load are converted to a voltage signal through a charge amplifier and data acquisition devices for output. For IEPE sensors, the charge amplifier is built in, which can make the sensing circuit go from high to low impedance and make it easy to set up.

**Figure 7:**
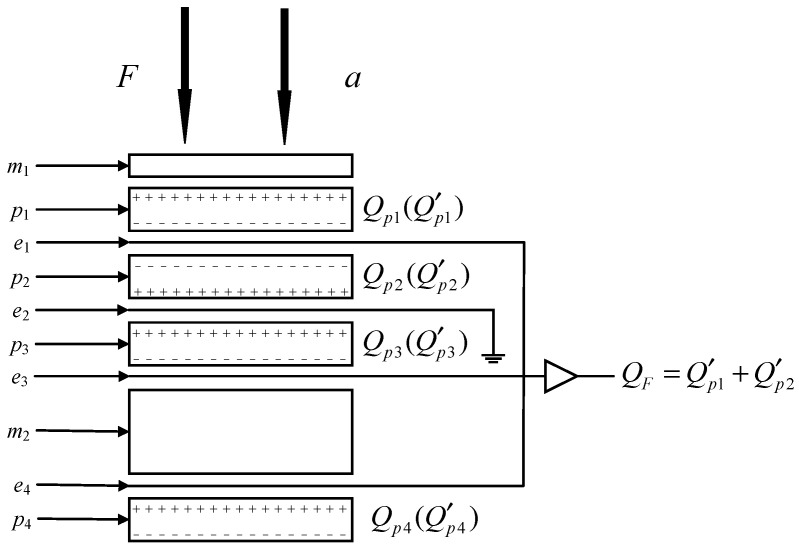
The schematic diagram of the acceleration compensation pressure sensor structure.

A pressure sensor with acceleration compensation is a good evolution for blast wave pressure measuring, since the explosion scenes are often accompanied by vibrations. By setting the polarity of quartz plates and selecting an appropriate block mass, the acceleration sensitivity can be eliminated or reduced. The structure consists of two mass blocks ($m_{1}$ for block of force and $m_{2}$ for acceleration compensation mass), four quartz plates ($p_{1}$, $p_{2}$, $p_{3}$ and $p_{4}$) and four piezoelectric electrodes ($e_{1}$, $e_{2}$, $e_{3}$ and $e_{4}$) which is shown in Figure 7. The $p_{1}$, $p_{3}$ and $p_{4}$ are connected with a wire jointly providing the output charge. For common used pressure sensor without acceleration compensation, the crystal group consists of $m_{1}$, $p_{1}$, $p_{2}$ and $e_{2}$. The $p_{1}$ and $p_{2}$ are set up in parallel. The added $m_{2}$, $p_{3}$, $p_{4}$, $e_{2}$, $e_{3}$ and $e_{4}$ are used for acceleration compensation. Actually, the charge sensitivity of the pressure sensor with and without acceleration compensation is equal to $2d_{ij}$ when the piezoelectric material is the same, where $d_{ij}$ stands for the piezoelectric constant. The details of the acceleration self-compensation mechanism of the piezoelectric pressure sensor can be seen in Ref. [17].

### 3.3. Modeling of Piezoelectric Pressure Sensors Under the Thermal Effect of Light

The influence of the thermal effect of light on piezoelectric pressure sensors can be simplified by adding a boundary heat source on the diaphragm surface. Assuming a 1 kg TNT charge with a thermal radiation efficiency of 3% (a conservative estimate for conventional explosives), the total thermal radiation energy is $E_{rad}=0.03\times Q_{v\left(\mathrm{TNT}\right)}\approx 123\mathrm{kJ}$, where $Q_{v\left(\mathrm{TNT}\right)}$ is explosion heat, a typical value is 4.23 × 10^3^ kJ/kg. At a distance R, neglecting atmospheric attenuation, the thermal exposure is:

(1) $$Q=\frac{E_{rad}}{4\pi R^{2}}=\frac{123000}{4\pi R^{2}}\approx \frac{10000}{4\pi R^{2}}\;{J/m}^{2}$$

The heat flux q(W/m^2^) can be estimated by q=Q/t. For 1 kg of TNT, the fireball duration t is approximately a few milliseconds. Assuming t=0.01 s, at a distance of 10 m, the heat flux is:

(2) $$q\approx \frac{10000}{10^{2}\times 0.01}=10000\;{W/m}^{2}$$

Therefore, the boundary heat flux is set to 10,000 W/m^2^ in the simulation. A preload force of 500 N is set above the diaphragm, and the boundary condition at the bottom of the sensor is roller support. The mesh is adopted in a physics-controlled mesh and the element size is extra fine. A transient study is carried out. The simulation duration is 10 s, and the time step is 0.01 s. This configuration is designed to simulate the output variation in the sensor under prolonged light exposure. The sensor boundary is set as thermal insulation, considering only the thermal conduction effect within the sensor. The heat convection outside the housing is ignored. The initial temperature is set at 20 °C.

**Figure 8:**
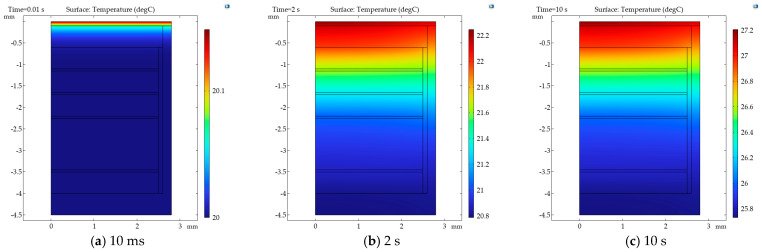
The temperature transfer cloud image of the simulation model.

The temperature cloud maps of the simulation results are shown in Figure 8. It can be observed that the boundary heat source is conducted from the sensor’s sensitive surface to the interior of the sensor.

**Figure 9:**
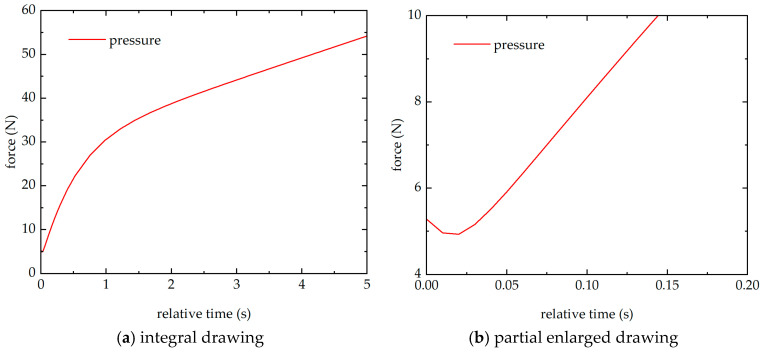
The algebraic sum of the pressure of the above two piezoelectric quartz plates in the simulation model.

In order to investigate the effect of thermal conduction on the sensor output, the algebraic sum of pressure applied to the two above piezoelectric quartz plates ($p_{1}$ and $p_{2}$) is plotted in Figure 9, with Figure 9b being a partial enlarged view. This algebraic sum of pressure corresponds to applying the same force to a single piezoelectric quartz plate. It can be observed that the pressure first slightly decreases and then increases significantly. Due to the leakage resistance in piezoelectric pressure sensors, they are unable to measure static pressure. Assuming the piezoelectric coefficient of the sensor remains constant, the output of the piezoelectric pressure sensor and the pressure on the piezoelectric crystal, as well as the time constant, should satisfy the following relationship $V\left(t\right)\propto F_{0}e^{-t/\tau }$. Therefore, the output of the piezoelectric pressure sensor exhibits a trend consistent with that shown in Figure 5, i.e., a decrease followed by an increase, and finally decaying to zero. As shown in Figure 5, the declining phase can be divided into two distinct stages. The first stage of the decline lasts approximately 200 ms, during which the sensor is likely in a non-steady-state heat transfer regime. Thereafter, as the interior of the sensor transitions into a steady-state heat transfer phase, the waveform becomes noticeably smoother. In fact, due to differences in internal structures, different piezoelectric sensors exhibit distinct outputs under thermal conditions, as demonstrated in the results shown in Figure 3 of Ref. [14].

In Figure 4, the peak value of P1 is 0.334 MPa, and the peak value of P2 is 0.375 MPa. The amplitude of the baseline drift in the blast wave front of P1 caused by the photothermal effect is 0.041 MPa. Therefore, when reading the peak pressure of a shock wave with baseline drift, the relative difference between the peak value and the amplitude of the baseline drop at the shock wave front should be considered. In Figure 4 the positive pressure duration of P1 is approximately 10 ms, while that of P2 is about 4 ms (with the baseline of the blast wave front serving as the zero-pressure reference). The photothermal effect leads to inaccuracies in reading the positive pressure duration of the blast wave. Measures should be taken to mitigate the impact of the photothermal effect on blast waveforms.

## 4. Discussion

In fact, Refs. [12,14,15,16,18] have all studied the impact of thermal shock on piezoelectric pressure sensors. Compared to piezoresistive pressure sensors, piezoelectric sensors are less affected under the same conditions [16]. On one hand, piezoresistive sensors experience coupled effects of both photoelectric and photothermal phenomena under illumination, whereas piezoelectric sensors are primarily influenced only by the photothermal effect. On the other hand, piezoelectric sensors possess leakage resistance, which allows parasitic charges generated by the photothermal effect to gradually decay over time. This contributes to the intuitive perception that piezoelectric sensors are less susceptible to light and heat. However, such influence should not be overlooked. When the photothermal effect or thermal shock acts on the sensitive surface of a piezoelectric pressure sensor, thermal stress is induced in the material due to thermal expansion. This thermal stress is subsequently applied to the piezoelectric quartz, leading to variations in the sensor’s output.

**Figure 10:**
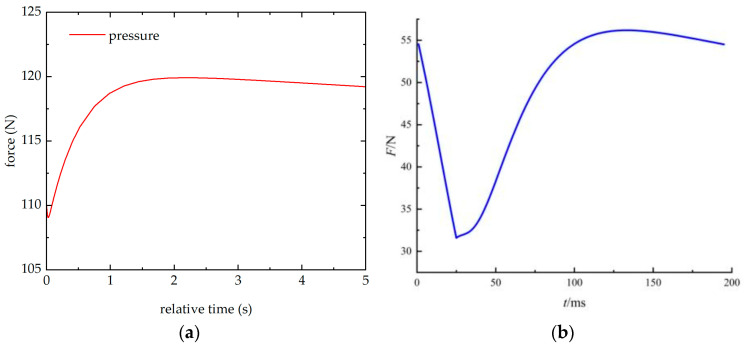
The pressure of the piezoelectric quartz plate 1 in the simulation model. (**a**) The simulation result of this paper, (**b**) the simulation results of Ref. [12].

Taking the pressure on the upper surface of piezoelectric quartz 1 in this study as an indicator, the influence of material thermal expansion on the sensor output was investigated. Figure 10a shows the pressure on the upper surface of piezoelectric quartz 1 in the above simulation model, while Figure 10b displays the pressure on the upper surface of piezoelectric quartz 1 from Ref. [12]. It can be observed that the variation trends of the pressure on the upper surface of piezoelectric quartz 1 are generally consistent in both cases, exhibiting an initial slight decrease, followed by an increase, and then another slight decrease. The difference lies in the fact that the simulation model in this study is subjected to a continuous external constant heat source, whereas the model in Ref. [12] is only exposed to a 30 ms thermal shock.

**Figure 11:**
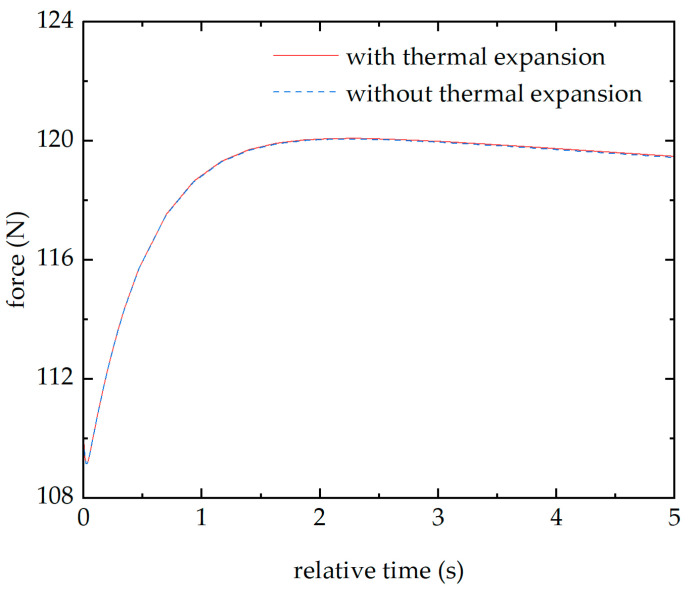
The forces of the piezoelectric quartz plate 1 with and without thermal expansion multiphysics fields in the simulation model.

In COMSOL Multiphysics, when the thermal expansion multiphysics is disabled, the pressure on the upper surface of the piezoelectric quartz plate 1 obtained from the simulation is shown in Figure 11. It can be observed that the pressure variation on the upper surface of the piezoelectric quartz plate 1 caused by thermal expansion is minimal. That is because the sensor’s mechanical structure restricts the quartz crystal, making it difficult to expand.

**Figure 12:**
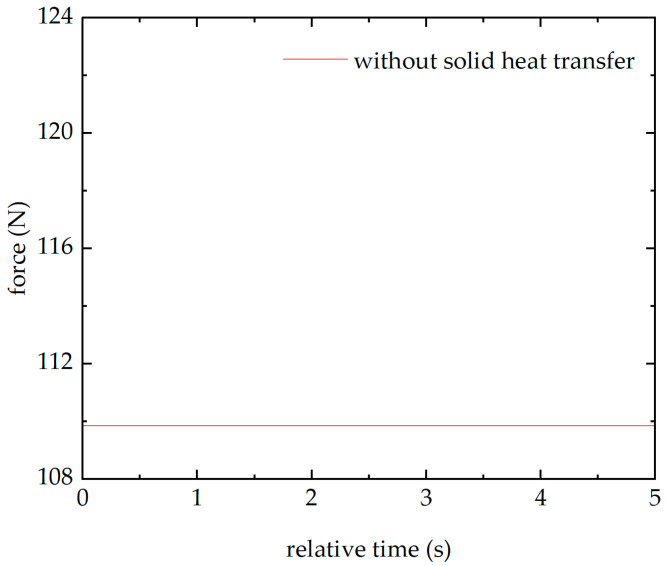
The force of the piezoelectric quartz plate 1 after removing the solid heat transfer field in the simulation model.

When the thermal expansion multiphysics is retained while the solid heat transfer field is disabled, the pressure on the upper surface of the piezoelectric quartz plate 1 is shown in Figure 12. It can be seen that the pressure remains essentially unchanged, indicating that conductive heat is the cause of the baseline drift phenomenon. Due to the constraints and external heat conduction, thermal stress occurs inside the sensor. This thermal stress is real and superimposes on the sensor’s output. The thermal stress is the fundamental cause of the baseline drift phenomenon.

**Figure 13:**
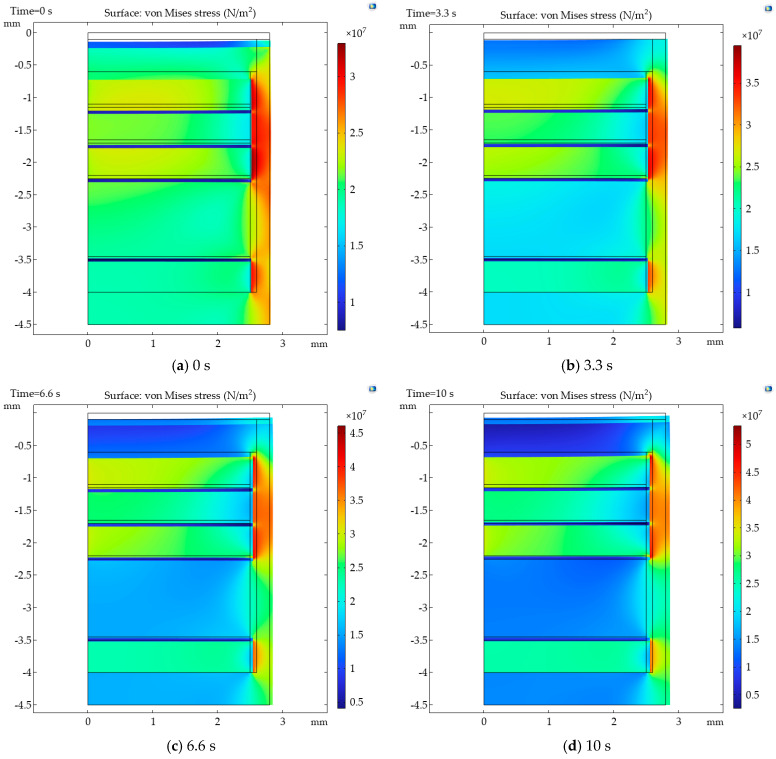
The von Mises stress nephograms of the simulation model.

The von Mises stress nephograms of the simulation model are shown in Figure 13, with a deformation scale factor of 200. It can be observed that the sensor expands outward over time. The edges of the force transmission block and the sleeve exhibit higher stress.

Furthermore, the heat acts on the target during an explosion in three forms, which arrive at the target in the following order: first, there is the photothermal effect induced by light; second, the thermal effect caused by the blast wave; and third, the heat generated by the explosion products. In Figure 3, the baseline drift phenomenon before the arrival of the blast wave is caused by the explosion light; after the blast wave arrives, the baseline drift phenomenon results from the combined effects of the photothermal effect of the explosion light and the thermal effect of the blast wave. When there are obstructions on the ground in the explosion field, some sensor-sensitive surfaces may not be exposed to the explosion light. This explains why not all piezoelectric pressure sensors exhibit baseline drift in explosion tests. The most effective way to suppress the baseline drift phenomenon is to add thermal insulation material outside the sensor. Refs. [14,15] discuss thermal insulation materials. When adding such materials, it is essential to determine their impact on blast wave pressure measurement.

## 5. Conclusions

This paper conducts a mechanistic analysis of the baseline drift phenomenon that occurs in piezoelectric pressure sensors during explosion testing. First, experiments confirmed that explosion light is the cause of the baseline drift phenomenon in piezoelectric pressure sensors. Subsequently, modeling and simulation of the piezoelectric pressure sensor were performed using the multiphysics simulation software COMSOL Multiphysics. The results determined that the photothermal effect induces changes in the internal temperature field of the sensor. Due to the constraints and external heat conduction, thermal stress occurs inside the sensor. This is identified as the fundamental cause of the baseline drift phenomenon in piezoelectric pressure sensors. The baseline drift phenomenon does not affect the interpretation of the blast wave overpressure peak but does impact the interpretation of the positive pressure duration of the blast wave. In explosion testing, it is essential to have a clear understanding of this phenomenon and implement necessary thermal insulation measures.

## Figures and Tables

**Table 1:** The geometrical parameters of the simulation model.

Element Name	Radii (mm)	Height (mm)	Material
Diaphragm	2.8	0.1	Invar
Block of Force	2.6	0.5	1Cr18Ni9Ti
Quartz Plate 1 to 4	2.5	0.5	Quartz (1949 IRE)
Electrode 1 to 4	2.5	0.05	Gold
Sleeve	Outer 2.6 Inner 2.5	1.05	1Cr18Ni9Ti
Housing	Outer 2.8 Inner 2.6	Outer 2.15 Inner 0.5	1Cr18Ni9Ti

**Table 2:** Thermophysical properties of materials.

Material	Coefficient of Thermal Expansion (C^−1^)	Thermal Conductivity (W·m^−1^·C^−1^)	Specific Heat Constant Pressure (J·kg^−1^·C^−1^)
Invar	1 × 10^−5^	11	515
1Cr18Ni9Ti	1.86 × 10^−5^	24.7	502
Quartz	5.5 × 10^−7^	10	800
Gold	1.42 × 10^−5^	317	129

## Data Availability

The data presented in this study are available on request from the corresponding author.

## Abbreviation

The following abbreviation is used in this manuscript:

IEPE	Integrated Electronics Piezo-Electric
